# Comparison of Ultrasound-guided Erector Spinae Plane Block Versus Rhomboid Intercostal Block for Perioperative Analgesia in Breast Cancer Surgery

**DOI:** 10.4274/TJAR.2026.252149

**Published:** 2026-06-26

**Authors:** Satish Kumar, Shagufta Naaz, Nishant Sahay, Sarfaraz Ahmad, Chandan Kumar Jha, Akhil VP, George Paul

**Affiliations:** 1 All India Institute of Medical Sciences New Delhi, Department of Anaesthesiology, Patna, India; 2 All India Institute of Medical Sciences New Delhi, Department of General Surgery, Patna, India; 3 Kerala University of Health Sciences, Government Medical College Idukki, Department of Anaesthesiology, Idukki, India; 4 St John’s Medical College, Department of Anaesthesiology, Bangalore, India

**Keywords:** Modified radical mastectomy, opioids analgesics, interventional ultrasonography, postoperative pain, fentanyl, nerve block

## Abstract

**Objective:**

Over 75% of women who have post-mastectomy reconstruction feel significant pain right away, and about 50% endure chronic pain. Thus, increasing the efficiency of postoperative pain management is crucial. Our study investigated the effects of ultrasound-guided erector spinae plane blocks (ESPB) and rhomboid intercostal blocks (RIB) on perioperative fentanyl use and pain scores in patients undergoing radical mastectomy surgery.

**Methods:**

This was a double-blind, randomised controlled trial conducted at a tertiary care hospital. Patients with breast cancer aged 18-70 years and American Society of Anaesthesiologists status I-II who were scheduled for unilateral modified radical mastectomy were included. They were randomly assigned to two groups. ESPB was performed in the ESPB group, and RIB was performed in the RIB group, using ultrasound guidance. Total postoperative fentanyl usage in the first 24 hours was the primary outcome indicator of the study. Intraoperative fentanyl requirements and numerical rating scale (NRS) scores at seven distinct time points were used as secondary outcome measures.

**Results:**

The difference between the mean of total postoperative fentanyl consumption in 24 hours in Group ESPB (2.67±0.68 µg kg^-1^) and Group RIB (3.68±1.22 µg kg^-1^) was statistically significant (t value: -4.183, df:66, *P* value: < 0.001). There was no difference in intraoperative opioid consumption between the groups (*P*=0.7). However, NRS scores were not significantly different between the ESPB group and the RIB group.

**Conclusion:**

Our study’s outcome demonstrates ultrasound-guided ESPB to be more effective than RIB in terms of lower perioperative fentanyl consumption.

**Main Points**

• This study investigated the effects of ultrasound-guided erector spinae plane block (ESPB) and rhomboid intercostal block (RIB) on perioperative fentanyl consumption and pain scores in patients undergoing radical mastectomy.

• Total postoperative fentanyl usage in the first 24 hours was the primary outcome indicator of the study.

• The difference in mean total postoperative fentanyl consumption over 24 hours was statistically significant between Group ESPB (2.67±0.68 µg kg^-1^)and Group RIB (3.68±1.22 µg kg^-1^) (*P* < 0.001).

• Our findings indicate that ultrasound-guided ESPB is more effective than RIB in reducing perioperative fentanyl requirements in breast cancer surgery.

## Introduction

The primary approach to treating breast cancer typically involves surgical interventions such as mastectomy, often supplemented with chemotherapy and radiotherapy.^1^ Due to the dense and complex nerve network in the breast region, inadequate management of acute postoperative pain can result in a transition to chronic pain. Notably, more than 75% of women undergoing post-mastectomy reconstruction experience significant acute pain, and nearly half continue to experience chronic pain.^2, 3, 4^ This underscores the critical importance of optimising postoperative pain control.

Regional anaesthesia techniques are foundational components of multimodal analgesia in breast cancer surgery.^5^ Previously, thoracic epidural was considered the gold standard in this regard. The PROSPECT guidelines for oncological breast surgery recommend paravertebral block (PVB), fascial blocks such as pectoral nerve block, and/or local anaesthetic infiltration for improved analgesia.^6^ However, conventional regional techniques such as PVB and thoracic epidural analgesia have notable limitations, including the technical complexity, the risk of hemodynamic instability, and the potential for bleeding or hematoma formation.^7^ Pectoral nerve block or local anaesthetic infiltration provides short-term pain relief.

In contrast, newer techniques such as the erector spinae plane block (ESPB) and rhomboid intercostal block (RIB) appear to offer promising alternatives. These blocks prevent entry into the paravertebral space, thereby minimising the risk of affecting the neuroaxis, major plexuses, and blood vessels. There is limited evidence on the analgesic efficacy of newer blocks, such as the ESPB and the RIB.

The ESPB is an interfascial technique for performing regional anaesthesia that is relatively simple to administer and provides effective postoperative analgesia in major thoracic procedures.^8^ In ESPB, local anaesthetic is injected deep to the erector spinae muscle at the T5-T6 level of the posterior chest wall. Some studies have reported that ESPB may affect the dorsal as well as ventral rami of multiple spinal nerves, with sensory spread often observed between approximately T3 and T9; however, the extent of spread varies across reports.^9^

The RIB, a more recently developed technique introduced by Elsharkawy et al.^10^ It involves injection of local anaesthetic into the fascial plane between the rhomboid major and the intercostal muscles at the T5-T6 level. Previous studies suggest that RIB can block dermatomes from T2 to T9 on the ipsilateral ventral and dorsal hemithorax, providing effective perioperative analgesia and reducing opioid requirements and associated side effects.^11^

Several studies of drug spread in ESPB have shown extension to the paravertebral and epidural spaces.^12, 13^ Hence, we expected that ESPB might provide more extensive analgesia in breast surgery as compared to RIB, where the spread of the drug is more peripheral. In the present study, we aimed to determine whether ultrasound-guided ESPB provided superior perioperative analgesia compared with RIB in breast cancer surgery.

## Methods

### Study Design and Ethics Committee Approval

Ethical approval for this study was obtained from the Institutional Ethics Committee of the All India Institute of Medical Sciences in Patna (approval no.: AlIMS/Pat/lEC/PGTh/Jan20/14, date: 25.01.2021). Following approval, it was planned as a randomized controlled trial at the All India Institute of Medical Sciences in Patna. The study was carried out in compliance with the World Medical Association’s Declaration of Helsinki after obtaining written informed consent from all patients prior to their enrolment. The trial was registered prospectively at the Clinical Trials Registry of India (CTRI/2021/03/031983). The study comprised patients scheduled for unilateral modified radical mastectomy surgery between 15 March 2021 and 14 March 2022 with American Society of Anaesthesiologists physical status I-II of age group 18-70 years and body mass index 18-24.9 kg m^2,-1^. The exclusion criteria were chronic opioid use, known allergy to local anaesthetics, procedure-site infection, previous mastectomy, and significant spinal or chest wall deformity.

### Anaesthetic Technique

All patients underwent a preoperative assessment, including a comprehensive history, physical examination, and relevant investigations. Participants were randomised to two groups: ESPB and RIB. Standard intraoperative monitoring electrocardiogram, non-invasive blood pressure (NIBP), and peripheral oxygen saturation (SpO_2_), was initiated upon arrival in the operating room. Baseline parameters, mean arterial pressure (MAP), heart rate (HR), and SpO_2_, were recorded as the last measurements just before induction. Intravenous Ringer’s lactate was administered at 15 mL kg^-1^ h^-1^.

Induction of general anaesthesia was performed with 2 µg kg^-1 ^fentanyl, 2 mg kg^-1^ propofol, and 0.1 mg kg^-1^ vecuronium, followed by endotracheal intubation. Anaesthesia was maintained with isoflurane (1-2%) in a 50% O_2_-air mixture, and end-tidal carbon dioxide was maintained between 30-35 mmHg. Vecuronium (0.02 mg kg^-1^) was administered every 30 minutes. Bispectral index was maintained between 40 and 60 by titrating the isoflurane concentration. Intraoperatively, 0.5 µg kg^-1^ fentanyl was administered when HR or MAP increased by >20% from baseline. NIBP was measured every 5 minutes. Baseline (preinduction), preincision (after administering the block and before incision), postincision, and any episode of hypotension were recorded. Hypotension (systolic blood pressure <90 mmHg), respiratory depression, and postoperative nausea and vomiting (PONV) were monitored. A senior anaesthesiologist, experienced in performing more than 20 ultrasound-guided fascial plane blocks, administered the allocated block but did not participate in patient assessment or data collection.

Prophylactically, ondansetron (4 mg), dexamethasone (8 mg), and paracetamol (15 mg kg^-1^) were administered 30 minutes before surgical closure. Injections of glycopyrrolate (0.01 mg kg^-1^) and neostigmine (0.05 mg kg^-1^) were used to reverse neuromuscular blockade.

### Group Allocation and Randomisation

Eligible patients were randomised using a computer-generated block randomisation with block sizes of 4 and 6. Allocation cards prepared using a random-number were sealed in opaque envelopes prepared by an independent investigator. Both participants and outcome assessors were blinded to group assignments. The anaesthesiologist performing the block was excluded from all postoperative assessments.

### Block Technique

### Group ESPB

**Figure 1:**
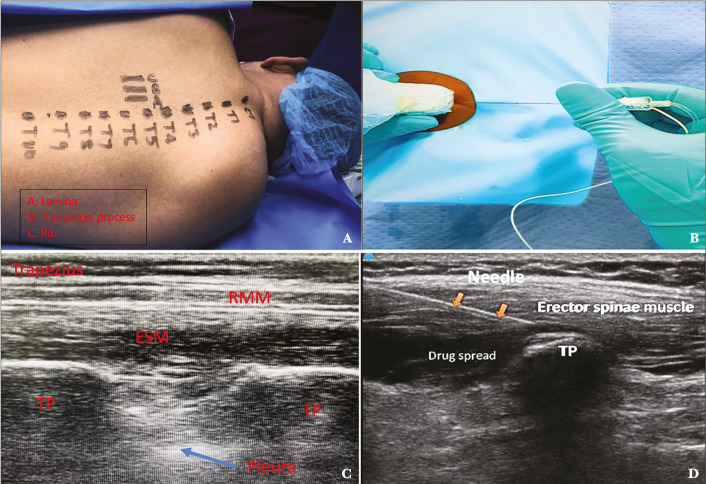
Erector spine plane block. A: Patient position and surface marking; B: Probe position and in-plane needling; C: Landmarks of ESPB from superficial to deep: Trapezius muscles, Rhomboid major, Erector spinae and T5 Transverse process; D: Placement of needle under ultrasound and spread of local anaesthetic drug. RMM, rhomboid major muscle; ESM, erector spinae muscle; TP, transverse process; ESPB, erector spinae plane block.

Following intubation, patients lie in the lateral position with the affected breast uppermost. The spinous processes from C7 to T5 were palpated, and the T5 level was marked. The trapezius, rhomboid major, erector spinae, and transverse processes were identified using a high-frequency linear ultrasound probe (M-Turbo, Fujifilm Sonosite Edge II, USA; 6-13 MHz) (Figure 1A). A 100-mm, 22-gauge needle (Stimuplex® Ultra 360®, B. Braun, Germany) was introduced in-plane 2-3 cm lateral to the midline until it contacted the T5 transverse process (Figure 1B and 1C). Confirming proper placement by injecting 5 mL of normal saline (hydro dissection), 25 mL of bupivacaine (0.25% ) was injected in aliquots, visualising its spread deep to the erector spinae muscle (Figure 1D).

### Group  RIB

**Figure 2:**
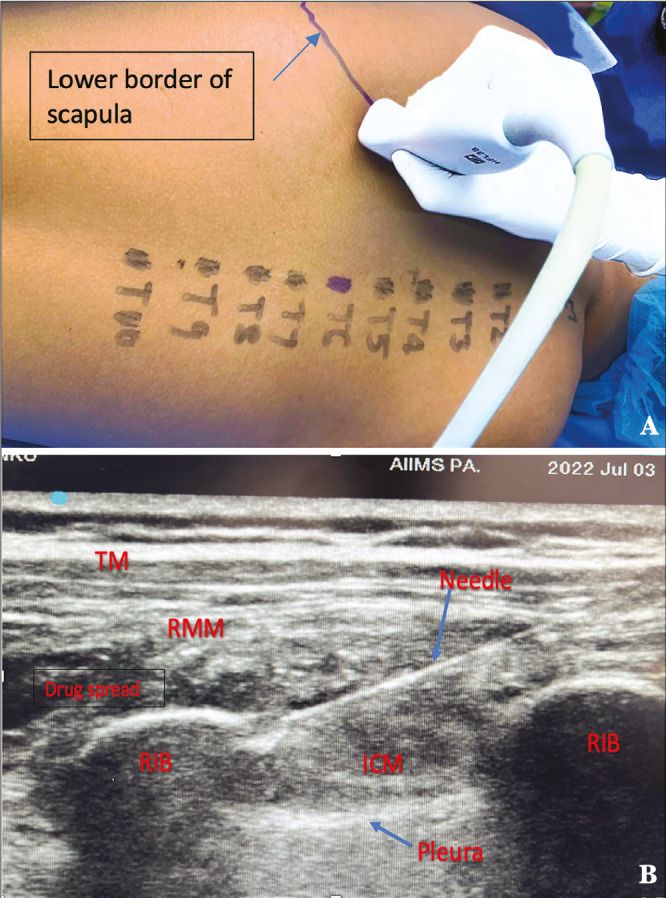
Rhomboid intercostal block. A: Placement of ultrasound probe for administration of rhomboid intercostal block; B: The Landmarks of rhomboid intercostal block and local anaesthetic injection under ultrasound view. TM, Trapezius muscle; RMM, Rhomboid major muscle; ICM, Intercostal muscle; RIB, rhomboid intercostal blocks.

Patients were positioned laterally in a similar manner. The arm on the upper side was placed across the chest to expose the triangle of auscultation, which is bordered by the trapezius, latissimus dorsi, and the scapula. Key anatomical landmarks (bounded by the trapezius, rhomboid major, intercostal muscles, rib, pleura, and lung) were identified using a 6-13 MHz linear ultrasound probe placed along the inferior scapular border (Figure 2A). A 100-mm, 22-gauge needle was introduced in-plane between the rhomboid major and the intercostal muscles. Following hydrodissection with 5 mL of saline, 25 mL of 0.25% bupivacaine was injected after negative aspiration (Figure 2B). Ultrasound was used to confirm appropriate spread. Patients were repositioned to the supine position for further surgical management. All other perioperative care followed the institutional protocols at All India Institute of Medical Sciences, Patna.

### Evaluation of Pain

Postoperative pain was assessed using the 11-point numerical rating scale (NRS), with 0 indicating no pain and 10 indicating the worst possible pain. NRS scores at 0, 1, 2, 4, 6, 12, and 24 hours were recorded postoperatively.

### Analgesia and Rescue Protocol

All patients received intravenous patient-controlled analgesia (PCA) on arrival in the post-anaesthesia care unit. The PCA was programmed to deliver 30 µg fentanyl boluses, with a 20 minute lockout and no background infusion. Time to first rescue analgesia and total fentanyl consumed over 24 hours were recorded. Intravenous paracetamol (15 mg kg^-1^) was administered every 8 hours. If NRS was >4 despite PCA fentanyl, intravenous tramadol (100 mg) was administered, and the dose was converted to fentanyl-equivalent doses for analysis.

### Outcome Measures

The primary outcome was total postoperative fentanyl consumption during the first 24 hours. Secondary outcome measures were: intraoperative fentanyl requirement; time to first rescue analgesia; NRS pain scores at defined intervals; intraoperative hemodynamic stability; and additional rescue analgesic requirements.

### Sample Size Calculation

Based on findings by Kumar et al.^14^where postoperative fentanyl consumption following RIB was 1.45±0.65 µg kg^-1^, a 30% reduction was deemed clinically significant for ESPB. With an alpha error of 5%, a power of 80%, and a 95% confidence interval, the sample size was estimated to be 68 (34 patients per group).

### Statistical Analysis

Data were initially recorded on a structured data-capture sheet , then compiled and analysed using IBM SPSS Statistics (version 25.0, student edition). Continuous variables with a normal distribution (e.g., age) were presented as mean ± standard deviation, whereas non-normally distributed continuous data were expressed as medians. Binary (categorical) variables were reported as absolute numbers and percentages.

Between-group comparisons of normally distributed continuous variables were performed using the unpaired Student’s t-test. To compare non-normally distributed continuous data or ordinal data between groups, the Mann-Whitney U test was applied. Repeated measures ANOVA was used for within-group analysis of changes across multiple time points.

## Results

**Figure 3:**
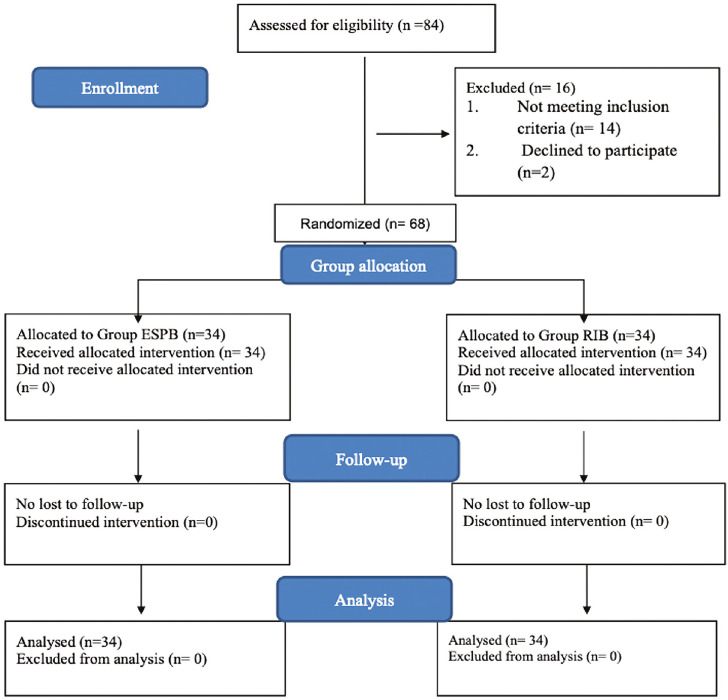
Consort chart. CONSORT flow diagram showing inclusion, randomization and exclusion of participants. ESPB, erector spinae plane block; RIB, rhomboid intercostal blocks.

Overall, 84 patients were assessed for eligibility. Fourteen of them did not fulfill the inclusion criteria, and two refused to participate. The remaining 68 patients were randomised and evenly allocated to two groups (Figure 3). Demographic characteristics of the two study groups were comparable, as shown in Table 1.

Postoperative total fentanyl consumption over 24 hours was normally distributed across groups, as verified through visual inspection of Q-Q plots. An independent t-test revealed that patients belonging to the ESPB group consumed significantly less fentanyl than those in the RIB group (mean difference, t=-4.183, df=66, *P* < 0.001). The calculated effect size (Cohen’s d=1.023) indicates a large clinical effect (Table 2).

In contrast, intraoperative fentanyl consumption did not follow a normal distribution across the groups. As shown in Table 2, there was no statistically significant difference in intraoperative fentanyl use between the ESPB and RIB groups.

Similarly, the time to first rescue analgesia was not normally distributed. Visual inspection of boxplots revealed no significant outliers. A Mann-Whitney U test showed no statistically significant difference in the time to first rescue analgesia between groups (U=508.5, z*=-*0.880, *P=*0.379) (Table 2). However, the frequency of rescue analgesic supplementation was significantly lower in the ESPB group than in the RIB group (U=895.0, z=4.203, *P *< 0.001).

The mean NRS pain scores did not differ significantly between the ESPB and RIB groups when analyzed using the Mann-Whitney U test, both at rest and during movement, as detailed in Tables 3A and 3B.

Repeated-measures ANOVA demonstrated that mean HR varied significantly across time points (F=96.870, *P *< 0.001). A significant decrease in HR between preinduction and preincision (after administration of blocks and before incision) and between preinduction and postincision was observed in Bonferroni-adjusted post-hoc pairwise comparisons (*P* < 0.001 for both comparisons). Preincision and postincision HR values did not differ significantly (*P*=1.000) (Table 4A). Similar changes were seen in MAP (Table 4B).

In our study, none of the patients reported adverse effects or complications, except for two patients in group EPSP and three patients in group RIB, all of whom experienced PONV.

## Discussion

This study compared the analgesic efficacy of the ESPB and the RIB in reducing perioperative opioid requirements in patients undergoing breast cancer surgery. Both blocks were administered safely under ultrasound guidance, with no observed procedural complications. ESPB produced greater opioid-sparing effects than RIB, as demonstrated by significantly lower 24-hour postoperative fentanyl consumption. This difference likely stems from their distinct anatomical spread patterns: ESPB facilitates paravertebral diffusion and, potentially, epidural diffusion, thereby offering broader dermatomal coverage, whereas RIB primarily targets the lateral cutaneous branches of intercostal nerves, resulting in more localised analgesia.^12, 13, 15^ However, the spread to the epidural and paravertebral spaces is less reliable and has not been found in all cases.^13^ Notably, intraoperative fentanyl requirements were similar between groups, indicating that the blocks provided comparable analgesia. An insignificant difference in intraoperative opioid requirement and in NRS scores could be related to other causes, such as different local anaesthetic absorption kinetics, rather than to a difference in anatomical spread between the two blocks.

The efficacy of ESPB has been well established in previous literature. Singh et al.^16^ reported enhanced analgesia with ESPB compared to controls. Altiparmak et al.^17^ observed a 35-40% reduction in tramadol use, while Kendall et al.,^18^ in a meta-analysis, reported an 8.84 mg reduction in intravenous morphine equivalents. ElHawary et al.,^19^ in a systematic review, further confirmed the effectiveness of ESPB for pain control following mastectomy. Although Gürkan et al.^20^ did not report any significant difference in NRS scores between ESPB and the control group, ESPB was associated with significantly lower morphine consumption compared with the control group in a study conducted by them. Most evidence—including randomised controlled trials, systematic reviews and meta-analyses—supports ESPB’s analgesic superiority in thoracic and breast surgeries.

In line with these findings, our results reinforce ESPB’s reliability in providing broad dermatomal coverage, producing significant reductions in postoperative opioid use, and delivering consistent analgesic efficacy across patient populations. These advantages validate ESPB as a potent regional anaesthetic technique for breast surgery.

RIB, although a relatively recent technique, has also demonstrated promising results. A meta-analysis by Chen et al.,^21^ including four randomised controlled trials with a total of 216 participants, showed that RIB significantly reduced acute postoperative pain and 24-hour opioid consumption compared to systemic analgesia. Cadaveric studies have confirmed RIB’s ability to achieve extensive craniocaudal and anteroposterior spread of dye, supporting its potential to effectively block intercostal nerves.^22^ Similarly, our study showed reduced pain scores, although the postoperative analgesic consumption and frequency of rescue analgesia were significantly higher with RIB than with ESPB (Tables 2 and 3A, 3B).

In our comparative analysis, ESPB demonstrated better opioid-sparing effects. However, both blocks yielded effective analgesia in the first 24 hours postoperatively, as reflected in low NRS scores both at rest and during movement. Thus, both blocks achieved clinically meaningful pain control throughout the postoperative period.

Zhang et al.^23^ compared the analgesic efficacy of ESPB, RIB, and serratus plane block (SPB) for video-assisted thoracic surgery. They found ESPB and RIB to be equally effective regarding postoperative sufentanil consumption and NRS scores, but both were superior to SPB. We found ESPB to have lower compared with RIB, although there was no significant difference in pain scores. The reason could be differences in the types of surgery. Both blocks could provide adequate analgesia in video-assisted surgery.

Jiang et al.^24^ compared ESPB, RIB, and SPB for pain management in modified radical mastectomy using 20 ml of 0.5% ropivacaine. They found RIB and ESPB to be equally effective, but both were superior to SPB in terms of opioid consumption. Çiftçi et al.^25^ found equal levels of pain relief with ESPB and RIB in breast-conserving surgery. They used 30 ml of 0.25% bupivacaine. These results could differ from our study, in which ESPB was found to be superior to RIB with respect to analgesic consumption, because different local anaesthetics, volumes, or concentrations were used in that study.

Hemodynamic outcomes were comparable between groups. Both ESPB and RIB maintained stable intraoperative cardiovascular parameters, with significant reductions in HR and MAP from baseline following block administration (*P* < 0.05); intergroup analysis revealed no significant differences (Table 4A, 4B). This indicates that both blocks were effective. Importantly, neither technique resulted in clinically significant hypotension, underscoring its cardiovascular safety profile.

Beyond analgesia, neither group exhibited opioid-related side effects such as constipation and pruritus, suggesting potential for enhanced postoperative recovery. No patient experienced respiratory depression or splinting due to pain, which further supports the utility of regional blocks in preserving respiratory function. The incidence of PONV was similar in both groups. Prior research by Chen et al.^21^ also reported a 20% absolute risk reduction in respiratory complications with RIB compared to systemic analgesia.

Crucially, no procedural complications such as pneumothorax, infection, or local anaesthetic systemic toxicity were encountered in our study. This outcome likely reflects the use of ultrasound guidance, adherence to strict aseptic technique, and the procedural expertise of the anaesthesiologist performing the block.

### Study Limitations

Despite these strengths, the study is not without limitations. A primary limitation of the study was that hemodynamic parameters were the sole trigger for analgesia, and no objective nociception monitoring, such as the Surgical Plethysmographic Index or Analgesia Nociception Index, was used. A fixed concentration of bupivacaine (0.25%) was used in all patients, precluding exploration of dose-response relationships at other doses. The study population was relatively homogeneous and limited to a single centre, which may limit the generalizability of the findings to broader age groups and different body mass index categories. Additionally, alternative local anaesthetic agents and combinations of volumes were not evaluated, thereby limiting the pharmacologic scope. We could not assess the dermatomal level of sensory block achieved by these two blocks because the patients were under general anaesthesia when the blocks were performed; this is another limitation of this study. We did not collect data on subjects receiving HR-lowering drugs, such as beta-blockers. We did not compare the comorbidities or patients taking antihypertensive medications between the groups. These also add to the limitations of this study. A limitation of the study is that we did not include a control group that received no block or a sham block.

## Conclusion

Our study demonstrates that ultrasound-guided ESPB results in significantly lower perioperative fentanyl consumption and less frequent analgesic supplementation than RIB. While both regional blocks effectively reduce pain in patients undergoing breast cancer surgery, ESPB provides better postoperative analgesia with a modest reduction in opioid consumption. These findings support the preferential use of ESPB to optimise postoperative analgesia in this surgical population.

## Ethics

**Ethics Committee Approval:** Ethical approval for this study was obtained from the Institutional Ethics Committee of the All India Institute of Medical Sciences in Patna (approval no.: AlIMS/Pat/lEC/PGTh/Jan20/14, date: 25.01.2021).

**Informed Consent:** All patients provided written informed consent prior to enrollment.

## Figures and Tables

**Table 2. Total Fentanyl used Perioperatively among the Groups:** 

-	**Group ESPB** **n = 34**	**Group RIB** **n = 34**	***P* value**
Total fentanyl used intraoperatively (µg kg^-1^)	2.07^#^	2.15^#^	0.716*
Postoperative fentanyl consumption in 24 hours (µg kg^-1^)	2.67±0.68^$^	3.68±1.22^$^	<0.001**
Time to first rescue analgesia (hours)	6 (3-8)^#^	5 (2-8)^#^	0.379*
Frequency of rescue analgesia required	3 (2-4)	4 (2-5)	<0.001*

^#^: median; *: Mann-Whitney U test; ^$^: mean *±* SD: **: unpaired t-test

EPSP, erector spinae plane block; RIB, rhomboid intercostal block; SD, standard deviation

**Table 3A. NRS at Rest Score among the Groups:** 

-	**Group ESPB**	**Group RIB**	-
**Time intervals (hours)**	**Median (IQR)**	**Median (IQR)**	***P* value^#^**
T0	1 (1.00)	1 (1.00)	0.579
T1	2 (0.00)	2 (0.00)	0.449
T2	2 (1.00)	1 (1.00)	0.229
T4	1 (1.00)	2 (1.00)	0.510
T6	1 (1.00)	1 (1.00)	0.498
T12	1 (1.00)	1 (1.00)	0.883
T24	1 (0.00)	1 (0.00)	0.832

^#^: Mann-Whitney U test

EPSP, erector spinae plane block; RIB, rhomboid intercostal block; IQR, interquartile range; NRS, numerical rating scale

**Table 3B. NRS Score at Movement among the Groups:** 

-	**Group ESPB**	**Group RIB**	***P *value^#^**
**Time intervals (hours)**	**Median (IQR)**	**Median (IQR)**	-
T0	2 (0.00)	2 (1.00)	0.742
T1	2 (1.00)	2 (1.00)	0.675
T2	2 (1.00)	2 (0.00)	0.071
T4	2 (0.00)	2 (1.00)	0.322
T6	2 (1.00)	2 (1.00)	0.765
T12	1 (0.00)	1 (1.00)	0.353
T24	1 (1.00)	1 (1.00)	0.239

^#^: Mann-Whitney U test

NRS, numerical rating scale; EPSP, erector spinae plane block; RIB, rhomboid intercostal block; IQR, interquartile range

**Table 4A. Post-hoc Pairwise Comparisons for Intraoperative Heart Rate:** 

**(I) time**	**(J) time**	**Mean difference (I-J)**	*P* value*
HR1	HR2	16.191*	<0.001
	HR3	16.868*	<0.001
HR2	HR3	0.676	1.000

*: Repeat measure analysis of variance; I, Column 1; J, Column 2; HR1, preinduction heart rate; HR2, preincision heart rate; HR3, postincision heart rate

**Table 4B. Post-hoc Pairwise Comparisons for Intraoperative Mean Arterial pressure:** 

**(I) time**	**(J) time**	**Mean difference (I-J)**	*P* value*
MAP1	MAP2	15.462*	<0.001
	MAP3	18.809*	<0.001
MAP2	MAP3	3.382	0.223

*: Repeat measure analysis of variance; I, Column 1; J, Column 2; MAP1, preinduction mean arterial pressure; HR2, preincision mean arterial pressure; HR3, postincision mean arterial pressure

**Table. 1 Comparison of Demographic Variables Between the Groups (Group ESPB vs. Group RIB):** 

-	**Group ESPB n = 34**	**Group RIB n = 34**	***P* value**
Age (years)	54.32±9.58^#^	54.09±9.634^#^	0.920*
Weight (kg)	51.21±4.73^#^	53.27±6.02^#^	0.121*
Height (cm)	151.32±2.77^#^	152.15±2.94^#^	0.239*
BMI (kg m^2-1^)	22.33±1.90^#^	22.81±2.04^#^	0.312*
ASA I:II	14:20	16:18	0.625**
Duration of surgery (min)	140.0 (110-180)^ ##^	140.0 (110-160)^ ##^	0.475***

^#^: mean ± SD; ^##^: median (IQR); *: unpaired t-test; **: chi-square test; ***: Mann Whitney U test

EPSP, erector spinae plane block; RIB, rhomboid intercostal block; ASA, American Society of Anaesthesiologists; BMI, body mass index; IQR, interquartile range

## References

[1] Manoharan N, Nair O, Shukla NK, Rath GK (2017). Descriptive epidemiology of female breast cancer in Delhi, India.. Asian Pac J Cancer Prev.

[2] Fecho K, Miller NR, Merritt SA, Klauber-Demore N, Hultman CS, Blau WS (2009). Acute and persistent postoperative pain after breast surgery.. Pain Med.

[3] Beederman M, Bank J (2021). Post-breast surgery pain syndrome: shifting a surgical paradigm.. Plast Reconstr Surg Glob Open.

[4] Agarwal S, Bharati SJ, Bhatnagar S (2021). The comparison of the efficacy of ultrasound-guided paravertebral block versus erector spinae plane block for postoperative analgesia in modified radical mastectomy: a randomized controlled trial.. Saudi J Anaesth.

[5] Gerbershagen HJ, Aduckathil S, van Wijck AJ, Peelen LM, Kalkman CJ, Meissner W (2013). Pain intensity on the first day after surgery: a prospective cohort study comparing 179 surgical procedures.. Anesthesiology.

[6] Jacobs A, Lemoine A, Joshi GP, Van de Velde M, Bonnet F; PROSPECT Working Group collaborators#. PROSPECT guideline for oncological breast surgery: a systematic review and procedure-specific postoperative pain management recommendations. Anaesthesia. 2020;75(5):664-673..

[7] Gomaa M, Abd El-Hamid AM, Elbarbary DH, Elmeliegy MF (2020). Ultrasound-guided erector spinae block for postoperative analgesia in thoracotomy patients: a prospective, randomized, observer-blind, controlled clinical trial. Ain-Shams J Anesthesiol..

[8] Chin KJ, Pawa A, Forero M, Adhikary S (2019). Ultrasound-guided fascial plane blocks of the thorax: pectoral I and II, serratus anterior plane, and erector spinae plane blocks.. Adv Anesth.

[9] López MB, Cadórniga ÁG, González JM, Suárez ED, Carballo CL, Sobrino FP (2018). Erector spinae block. A narrative review.. Cent Eur J Clin Res.

[10] Elsharkawy H, Saifullah T, Kolli S, Drake R (2016). Rhomboid intercostal block.. Anaesthesia.

[11] Abd Ellatif SE, Ibrahim ES, Fathi HM (2025). Rhomboid intercostal versus serratus anterior plane block for analgesia after thoracodorsal artery perforator flap following partial mastectomy: a randomized controlled trial.. Pain Physician.

[12] Sørenstua M, Zantalis N, Raeder J, Vamnes JS, Leonardsen AL (2023). Spread of local anesthetics after erector spinae plane block: an MRI study in healthy volunteers.. Reg Anesth Pain Med.

[13] Lim H, Mathew C, Wong SN, Liu CW (2023). Anatomical insights into injectate spread after thoracic erector spinae plane block: a systematic review.. J Clin Anesth.

[14] Kumar A, Sinha C, Kumari P, Kumar A, Sinha AK, Kumar B (2020). Ultrasound guided rhomboid intercostal block: a pilot study to assess its analgesic efficacy in paediatric patients undergoing video-assisted thoracoscopy surgery.. Indian J Anaesth.

[15] Almesned S (2025). A comprehensive review on rhomboid intercostal block as postoperative analgesia in breast surgery.. The Open Anesthesia Journal.

[16] Singh S, Kumar G, Akhileshwar (2019). Ultrasound-guided erector spinae plane block for postoperative analgesia in modified radical mastectomy: a randomised control study.. Indian J Anaesth.

[17] Altiparmak B, Toker MK, Uysal Aİ, Kuşçu Y, Demirbilek SG (2019). Eficácia do bloqueio do plano do músculo eretor da espinha guiado por ultrassom para analgesia após colecistectomia laparoscópica: um estudo controlado randômico [Efficacy of ultrasound-guided erector spinae plane block for analgesia after laparoscopic cholecystectomy: a randomized controlled trial].. Braz J Anesthesiol.

[18] Kendall MC, Alves L, Traill LL, De Oliveira GS (2020). The effect of ultrasound-guided erector spinae plane block on postsurgical pain: a meta-analysis of randomized controlled trials.. BMC Anesthesiol.

[19] ElHawary H, Abdelhamid K, Meng F, Janis JE (2019). Erector spinae plane block decreases pain and opioid consumption in breast surgery: systematic review.. Plast Reconstr Surg Glob Open.

[20] Gürkan Y, Aksu C, Kuş A, Yörükoğlu UH (2020). Erector spinae plane block and thoracic paravertebral block for breast surgery compared to IV-morphine: a randomized controlled trial.. J Clin Anesth.

[21] Chen R, Su S, Shu H (2022). Efficacy and safety of rhomboid intercostal block for analgesia in breast surgery and thoracoscopic surgery: a meta-analysis.. BMC Anesthesiol.

[22] Elsharkawy H, Maniker R, Bolash R, Kalasbail P, Drake RL, Elkassabany N (2018). Rhomboid intercostal and subserratus plane block: a cadaveric and clinical evaluation.. Reg Anesth Pain Med.

[23] Zhang JG, Jiang CW, Deng W, Liu F, Wu XP (2022). Comparison of rhomboid intercostal block, erector spinae plane block, and serratus plane block on analgesia for video-assisted thoracic surgery: a prospective, randomized, controlled trial.. Int J Clin Pract.

[24] Jiang CW, Liu F, Zhou Q, Deng W (2021). Comparison of rhomboid intercostal nerve block, erector spinae plane block and serratus plane block on analgesia for modified radical mastectomy: a prospective randomised controlled trial.. Int J Clin Pract.

[25] Çiftçi B, Basım P, Güngör H, Alver S, Birzat EG, Atalay YO (2025). Comparison of postoperative analgesic efficacy between erector spinae plane block and rhomboid intercostal block in breast-conserving surgery and sentinel lymph node biopsy: a randomized non-inferiority clinical trial. Aǧrı..

